# Ophthalmic practice protocols during the COVID-19 pandemic – the Aravind way

**Published:** 2020-09-01

**Authors:** N Venkatesh Prajna

**Affiliations:** 1 Academic Director: Aravind Eye Hospital, Madurai, India.

**Protocols, training and regular meetings at senior level have shaped Aravind Eye Hospital’s response to the pandemic.**

**Figure:**
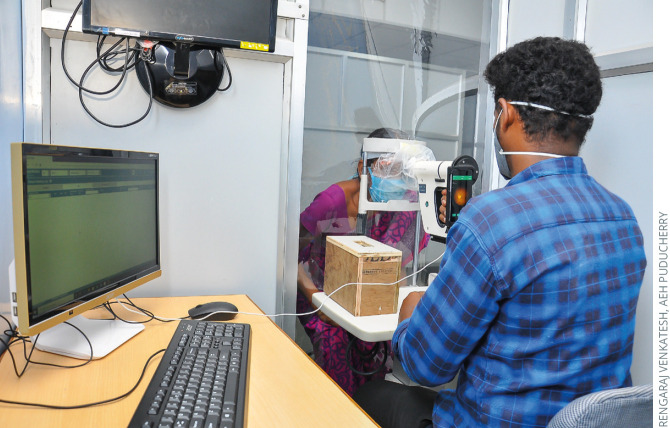
Transparent plastic screens protect health workers and patients at Aravind Eye Hospital. **INDIA**

On 25 March 2020, the government in India announced a country-wide lockdown. Aravind Eye Care System closed vision centres at once and stopped offering refraction, routine outpatient services, elective surgery, and community outreach activities. Tertiary centres and secondary centres remained open for emergencies, but eye donation activities ceased following a directive from the Eye Bank Association of India.

After the first few days of uncertainty, one of the first steps we took was to put policies and protocols in place and to plan staff training. The senior doctors across Aravind Eye Care System also started to meet regularly to come up with new protocols as and when the situation demands.

We put up posters in all Aravind hospitals about the importance of hand washing and social distancing, and taught staff members about the importance of personal protective equipment (PPE) and what they needed to use, depending on what work they were doing. We check and audit this every day.

**Figure:**
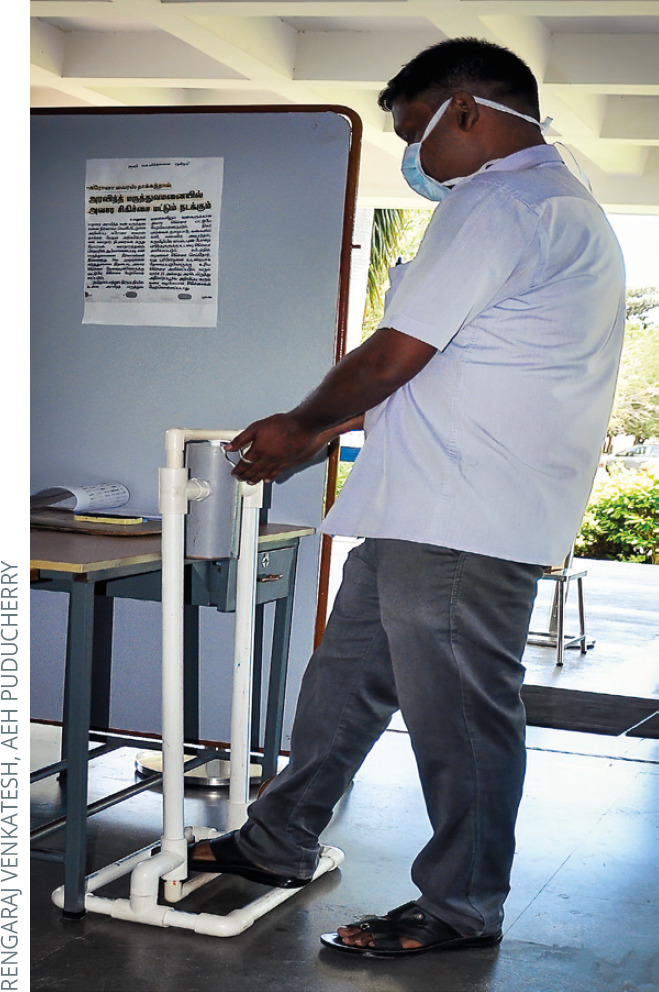
A locally made foot pump allows patients to use hand sanitiser without having to touch the bottle with their hands. **INDIA**

## Triaging

All patients and employees must undergo temperature checks using a thermal scanner, and everybody entering the hospital must wash their hands thoroughly at a separate handwashing basin. Health care providers and patients must wear a mask as standard practice, and no-one may go with patients.

We have set up a face-to-face triaging centre. Patients with emergency eye conditions, such as severe conjunctivitis, sudden loss of vision and significant trauma, are referred to a small, rotating team of doctors, refractionists and nurses. Surgery is limited to emergencies such phacolytic and phacomorphic glaucoma, corneal and orbital trauma and retinal detachment, and only essential investigations are performed.

## Communication with patients

Postoperative patients, patients on long-term medication and others can ring a dedicated call centre if they need advice. We have also set up a dedicated audio-visual tele-consultation system to enable patients to have a consultation with an ophthalmologist in real time. This has proved to be successful, and we are considering making this a more permanent and robust facility in future.

## Infection control practices

In addition to existing infection control practices, it is now standard practice to keep windows open to improve ventilation, clean door handles, chairs, slit lamps and computers regularly (using sodium hypocholorite (bleach) or benzalkonium chloride solution), and to disinfect hands (after hand washing) with alcohol-based hand sanitisers.

Intraocular pressure is measured by applanation tonometry only, and we clean tonometry tips and ultrasonography probes using bleach solution or alcohol swabs.

All slit lamps are fitted with a plastic shield barrier made from large x-ray plates or plastic files. Essential shops within the hospital, such the medical and optical shops, function with as few staff members as possible and offer only limited services.

The supply of protective masks, gloves and disinfectant is an ongoing challenge and is continuously monitored to ensure a smooth supply.

## Restarting services at Aravind

Aravind restarted its routine outpatient facilities in early May. Only 10% of patients are accessing the facilities at the time of writing due to issues with transport. Trauma, retinal detachment surgery and intravitreal injections are continuing as usual, but we are awaiting government guidelines before we start elective surgery, including cataract surgery. If routine testing for COVID-19 before surgery becomes mandatory, there will be significant delays as we do not have access to a private testing facility; the government hospital facilities are overwhelmed.

